# On the efficacy of procedures to normalize Ex-Gaussian distributions

**DOI:** 10.3389/fpsyg.2014.01548

**Published:** 2015-01-07

**Authors:** Fernando Marmolejo-Ramos, Denis Cousineau, Luis Benites, Rocío Maehara

**Affiliations:** ^1^Gösta Ekman Laboratory, Department of Psychology, Stockholm UniversityStockholm, Sweden; ^2^School of Psychology, University of OttawaOttawa, ON, Canada; ^3^Department of Statistics, Institute of Mathematics and Statistics, University of São PauloSão Paulo, Brazil

**Keywords:** Ex-Gaussian, reaction times, normality tests, outliers

## Abstract

Reaction time (RT) is one of the most common types of measure used in experimental psychology. Its distribution is not normal (Gaussian) but resembles a convolution of normal and exponential distributions (Ex-Gaussian). One of the major assumptions in parametric tests (such as ANOVAs) is that variables are normally distributed. Hence, it is acknowledged by many that the normality assumption is not met. This paper presents different procedures to normalize data sampled from an Ex-Gaussian distribution in such a way that they are suitable for parametric tests based on the normality assumption. Using simulation studies, various outlier elimination and transformation procedures were tested against the level of normality they provide. The results suggest that the transformation methods are better than elimination methods in normalizing positively skewed data and the more skewed the distribution then the transformation methods are more effective in normalizing such data. Specifically, transformation with parameter lambda -1 leads to the best results.

## INTRODUCTION

Reaction times (RTs) have been a privileged measure of behavior in experimental psychology allowing an estimation of the duration of cognitive processes and inference of the likely cognitive process (see Donders et al., 1969). Hence, their understanding and proper analysis is essential. It is known that reaction time data are positively skewed, and therefore are not normally distributed. As Olivier and Norberg (2010) argue, commonly used statistical tests are not appropriate for the analysis of RT data since RTs are (in most cases) non-normally distributed. Yet, most researchers rely on parametric tests (primarily ANOVA) to analyze reaction times data despite these tests assumptions are not met with RT data. More specifically, they require variables to be normally distributed within conditions and have homogeneous variances between conditions in order to give unbiased results (e.g., Calkins, 1974; Marmolejo-Ramos and González-Burgos, 2013). Even small violations of those assumptions can lead to biased results from the tests (see Wilcox, 1998). To meet these conditions, many researchers transform the data and/or search for maverick data points. The aim of this paper is to compare various procedures that assist in normalizing data via power transformations and outlier elimination procedures.

### REACTION TIME DISTRIBUTIONS AND METHODS TO DEAL WITH OUTLIERS

Reaction time distributions are characterized by a positive skew. Many explanations have been proposed to explain this near universal finding (with one exception; Hopkins and Kristofferson, 1980, who found symmetrical RT distributions). The first explanation from McGill (1963), argued that observable RTs are caused by two processes operating in succession. The first is a central decision mechanism whose distribution is highly skewed (exponential distribution). This mechanism is related to an accumulation of information processes whose activation times have a rate of accumulation τ ms^-1^. These assumptions are based on neurological studies of single cell firing patterns (see e.g., Langlois et al., 2014) showing that for a small threshold, the resulting distribution is very skewed and well-described by an exponential distribution. The second process is responsible for response selection and motor execution. This second process is presumably affected by many factors and therefore (owing to the central limit theorem) results in a normal distribution. The sum of these two sets of time has a distribution described by a convolution of a Gaussian distribution and an exponential distribution known as an Ex-Gaussian distribution. Hohle (1965), Ratcliff and Murdock (1976), Hockley (1984), and Heathcote et al. (1991), among others, fitted this distribution to RTs and found a generally good fit.

In other words, a simple cognitive task starts at the level of the perceptual processes. Light travels to the retina (negligible time), activating the cones and rods on the retinae and transducing the signal through the visual brain areas (V1, V2, etc.). Following perception and up to a semantically meaningful percept, there is the decision process presumably occurring in the frontal lobes. A decision is then followed by activations sent for response selection and down to the motor areas and spinal cord triggering a muscular response, which puts pressure on a response key. Overall, a simple decision involves a chain of signal transformation through a dozen specialized brain areas each adding to the observed latency. The total processing chain can be subdivided into three stages: perception, decision and response selection, and motor response. Based on the assumption that the time taken by each brain area adds up to the total response time observed by the apparatus, and knowing that manipulating the difficulty of the decision without altering the perceptibility of the stimuli and without altering the motor response complexity can affect skew, it can be hypothesized that (1) perception processes add up to a total perception time; (2) response selection and motor response processes add up to a total response time, and (3) the balance leads to a decision time. Although the time taken by the perceptual processes are unknown, owing to the Central limit theorem, if multiple processes with unknown times are added to obtain a total time then the resulting perception time should be normally distributed. This same principle applies to the response selection and motor response stage.

In recent years, though, some authors have questioned the additivity assumptions. Under an alternative view of the chain of processing, the brain areas send activations continuously and related areas react when a critical amount of activations have been received. Hence, each area is not operating in isolation. Thus, violating the independence of operation of each sub-process implicit in the additivity assumption. Theorems analogous to the Central limit theorem suggest that the resulting perception time should be log-normally distributed in this scenario (Ulrich and Miller, 1993; Mouri, 2013). Finally, as the decision processes are based on just a few sub-processes, asymptotic theorems cannot be invoked and this stage should preserve its highly skewed characteristic at the level of latency.

Other explanations have subsequently been proposed to explain the skew in RTs. Ulrich and Miller (1993) suggested that response times may be caused by a cascade of events (following McClelland, 1979, cascade model). This model predicts a distribution called the Log-Normal (also see West and Shlesinger, 1990) whose shape is indistinguishable from the EGd (Chechile, personal communication). Raab (1962), followed by LaBerge (1962) and Pike (1973), instead proposed a race model where brain signals compete with each other to be the first to trigger a response (recent documentation includes Rouder, 2000; Miller and Ulrich, 2003; Cousineau, 2004). These models all suggest that the Weibull distribution should be the distribution of RTs (see also Schwarz, 2001 for a variation of this idea). The Weibull and Ex-Gaussian/Log-Normal can be in principle distinguished, but this requires a lot of RTs per conditions (more than 100), uncontaminated by practice effects, fatigue effects, etc. Nevertheless, the true distribution of RT may also (more likely) be none of the above.

**FIGURE 1:**
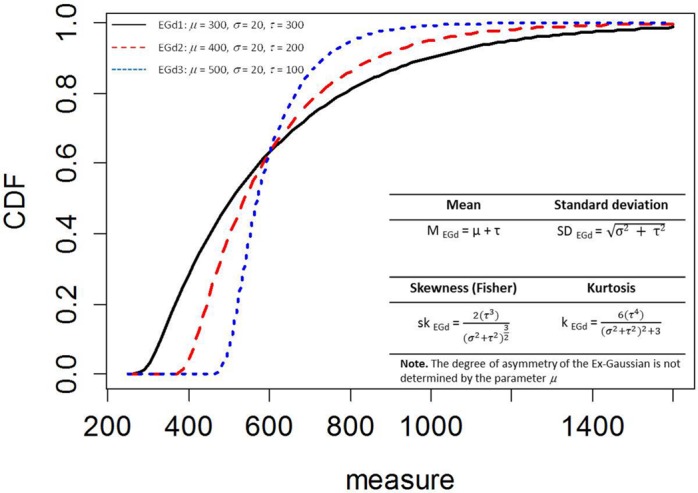
**Empirical density functions (ECDF) of three Ex-Gaussian distributions (EGDs) with different parameters.** Bottom right inset shows the moments of the EGD. PDFs of these distributions can be seen in Figure 3 in Marmolejo-Ramos and González-Burgos (2013).

In what follows, we assume that the EGd is a convenient way to characterize RTs much like statisticians assume the normal distribution. Furthermore, the literature indicates that the EGd is the distribution most broadly explored. A comprehensive characterization of EGds can be found in Marmolejo-Ramos and González-Burgos (2013). The parameters of an EGd are represented by a mean (μ), a SD (σ), and an exponential factor (*τ*). The mean and the SD represent times from the normally distributed stage of processing, whereas the exponential factor represents times from the exponentially distributed stage of processing (see McAuley et al., 2006). The mean of an EGd can be inferred from its parameters, as well as its standard variation. The EGd’s SD and exponential factor can be used to estimate its third (skewness) and fourth (kurtosis) central moments (see **Figure 1**).

It is a common practice in psychological research that measures RT data to deal with observations which fall very far from a group’s mean. Such observations are possibly the product of participants’ lack of attention (very long RTs) or overly fast guesswork (very fast RTs). However, it may also be caused by random fluctuations in internal thresholds. These outliers deeply affect the estimation of the data’s central tendency (see Whelan, 2008; Baayen and Milin, 2010; Cousineau and Chartier, 2010). Although for some researchers, outliers are not influential in data sets (see Orr et al., 1991; Lance et al., 1996), most psychologists (e.g., Judd et al., 1995) and statisticians (e.g., Beckman and Cook, 1983) agree that outliers affect parameter estimation. Outliers are usually dealt with by using pre-determined criteria; mainly via SD cut-offs (e.g., eliminating observations above and/or below 2 SD, see Cowles and Davis, 1982) or data re-expressions (e.g., transforming the data into logarithms, see Bland and Altman, 1996). Additionally, a combination of these approaches has been proposed (e.g., transforming the data and then removing outliers or vice versa, see Marmolejo-Ramos and Matsunaga, 2009).

Under the 2 SD procedures, researchers remove observations which fall ± 2 SD from a participant’s mean in a particular condition (see Ratcliff, 1993; for an example of this application see Bertels et al., 2010). This procedure trims long tails in RT distributions on a subject per condition basis, but at the cost of leaving experimental conditions with an unequal number of trials. Although SD cut-off leads to an underestimation of the population’s RT, such a biased estimation appears to depend on sample size (Perea, 1999). In addition, the cut-off values are symmetrical about the mean but the data are not. Hence, it is more likely that high outliers will be removed, resulting in a systematic bias to reduce the observed mean. To minimize overestimation bias, some researchers have proposed adjustments for a number of SD according to sample size (see Van Selst and Jolicoeur, 1994; Thompson, 2006; see also Table 2 in Cousineau and Chartier, 2010). Nevertheless, in practice most researchers rarely adjust SD cut-off according to sample size using a 2 SD, 2.5 SD, or even a 3 SD cut-off criterion instead (for examples, of each see Havas et al., 2007; Bertels et al., 2010; Otte et al., 2011; respectively, see Leys et al., 2013; for a review).

The SD cut-off is the most commonly used procedure to deal with outliers in RT research. However, advances in statistics suggest the use of more robust methods to deal with outliers. One such approach derives from multivariate outlier detection methods and is called the minimum covariance determinant (MCD) method. This method aims to estimate the best subset of normally distributed points in a data set which are clustered in an ellipsoid with the smallest volume (or minimum covariance matrix). The computations of the MCD rely on Mahalanobis distances and robust estimators of multivariate location (see Rousseeuw and van Driessen, 1999). Although the MCD method is primarily designed to deal with multivariate data, it does not preclude it from being applied to univariate data.

Another approach to deal with non-normality is data transformations. With this procedure all observations are retained but they are re-expressed using a different, non-linear, scale that improves normality of the data (see Osborne, 2002; Olivier and Norberg, 2010). RT data can be re-expressed into logarithms (for an example of this application see Markman and Brendl, 2005), square-roots (for an example see Moran and Schwartz, 1999), and inverse (for an example see Moss et al., 1997). A well-known transformation that achieves all these re-expressions is the Box–Cox transformation. In this transformation, the selection of a particular parameter known as lambda, is accompanied by a (restricted) log-likelihood statistic that signals the best parameter needed in order to achieve the highest normality (see Olivier and Norberg, 2010). Thus, specific lambda parameters have been associated with the inverse (lambda = -1), logarithmic (lambda = 0), and square-root (lambda = 0.5) transformations and previous studies have suggested that the inverse transformation has a strong normalization effect (see Ratcliff, 1993).

A simulation study in which the normalization power of the Box–Cox transformations and elimination procedures are tested against a particular type of skewed distributions is yet to be done. As to the Box–Cox method, it would be useful to see how other transformation parameters could improve the normality of EGds. Thus, the intermediate parameters -0.5 – are worth testing since it can be seen as a trade-off between an inverse (i.e., -1) and a logarithmic (i.e., 0) transformation. The present simulation study aims to test the power of these outlier elimination and transformation methods to normalize EGds of different parameters and sample sizes. The results will indicate the most effective methods when dealing with positively skewed distributions.

## MATERIALS AND METHODS

### VALIDATION OF AN ALTERNATIVE SIMULATION METHOD AND A COMPREHENSIVE APPROACH TO THE ASSESSMENT OF NORMALITY IN NON-NORMAL DISTRIBUTIONS

In order to determine how various outlier elimination and transformation methods can improve the normality of data sampled from EGds, it is necessary to first check the normality of the EGds before applying these methods. A typical approach is to estimate the power of normality tests against non-normal distributions. Under this approach it is traditional to (i) Compute the Critical values (CVs) of one or more normality tests against a *N*(0,1) of different sample sizes, and to (ii) Use those CVs as cut-off points to reject normality in non-normal distributions of the same sample sizes used in the simulations (see Marmolejo-Ramos and González-Burgos, 2013, for a detailed explanation).

The power of a normality test relies on the number of times the test correctly rejects normality. We note hereafter the proportion of rejection of normality as PoR. A high PoR (e.g., a PoR close to 1) signifies that the distribution being tested is highly non-normal, whereas a low PoR indicates otherwise (e.g., a PoR close to 0). On the other hand, all tests should show PoRs hovering around 0.05 (as α = 0.05) when tested against a normally distributed set of data regardless of the sample size (e.g., Romão et al., 2010). Such a situation is to be expected since normality tests should have a low probability of incorrectly rejecting the hypothesis that a *N*(0,1) is normal, and that probability should be close to the nominal level used in the study (the α level). In sum, normality tests should have a low PoR, against normal distributions and have a high power, or a high PoR, against non-normal distributions.

In a recent study, Marmolejo-Ramos and González-Burgos (2013) studied the power of various normality tests against three EGds and other non-normal distributions such as the Weibull (2,1) and Log-Normal (0,1). Their results not only replicated the comprehensive results reported by Romão et al. (2010) regarding the Weibull and Log-Normal distributions, but also found that of all the normality tests studied, the Shapiro–Wilk (SW) was the test with the highest power against EGds. For instance, using the CVs approach described above, these researchers found that the SW test has a power of approximately 0.45 when dealing with EGds with parameters μ = 300, σ = 20, and τ = 300 when the sample size was 10. When the sample size was 10 and the parameters of the EGds were μ = 400, σ = 20, and τ = 200 and μ = 500, σ = 20, and τ = 100, the powers of the SW test were just below 0.45 and between 0.35 and 0.40, respectively (see Figure 4, in their study for more detailed results).

The present study features an alternative approach in which the *p*-value associated with a normality test is used. Marmolejo-Ramos and González-Burgos (2013, footnote 5) argue that in simulation studies, *p*-values are not mandatorily calculated as they are calculated in statistical packages. That is, statistical packages rely on theoretical distributions, while simulation studies rely on empirical distributions. The present study uses the *p-*value associated with a normality test, as given in statistical packages, to validate its usage as an alternative way to measure the PoR given by a normality test for a certain distribution. The PoR results obtained via the *p-*value are simply the proportion of times a normality test gives *p*-values below, and is not equal to, a chosen alpha level, e.g., α = 0.05, when tested against a certain distribution of a particular sample size. Thus, if the *p-*value approach proposed herein is effective, it should be able to replicate or approximate the results found by Marmolejo-Ramos and González-Burgos (2013) regarding the power of SW test against various EGds.

To validate the *p-*value approach, a simulation study was performed to test the power of the SW against the same EGds described above, when sample size was 10, and with an alpha level of 0.05. Additionally, the present simulations implement the method proposed by Marmolejo-Ramos and González-Burgos (2013) consisting of iterating (i) each simulation (*s*) a set number of times and estimating measures of central tendency *(a)* and dispersion (SD) across iterations. Thus, these parameters were: *i* = 30, *s* = 20’000, and *a* = the Mean (and its ±1 SD). That is, each run of 20’000 simulations was iterated 30 times and for each vector containing 30 iterations, the mean PoR, the mean *p*-value and their associated SDs were estimated. More importantly, the results of the iterations are amenable to formal statistical analysis in order to determine the main effects of and interactions between the variables included in the simulations. The results indicate that the proposed *p*-value method does replicate the results obtained by Marmolejo-Ramos and González-Burgos (2013); PoR: *M*_EGd1_ = 0.427 (SD = 0.003), *M*_EGd2_ = 0.413 (SD = 0.003), and *M*_EGd3_ = 0.354 (SD = 0.003); *p*-value: *M*_EGd1_ = 0.178 (SD = 0.001), *M*_EGd2_ = 0.188 (SD = 0.001), and *M*_EGd3_ = 0.232 (SD = 0.001), as determined by the SW normality test when *n* = 10.

As suggested above, it is essential to determine the status of non-normal EGds before any outlier elimination or transformation method is applied. As is traditional in simulation studies of normality tests (see Romão et al., 2010; Alizadeh Noughabi and Arghami, 2011; Yap and Sim, 2011), the PoR of a particular normality test is computed for a certain distribution of a certain sample size. Indeed, this is the usual means of evaluating the normality of a data set, i.e., usually researchers use the SW or the Kolmogorov–Smirnov test (KS) to determine what the normality of a data set is (see Marmolejo-Ramos and González-Burgos, 2013). However, as has been shown through simulation studies of normality tests, some tests have higher power than others in determining normality, and the type of distribution being tested plays a role in this (Engmann and Cousineau, 2011). Thus, relying on a sole normality test could be problematic in that it is difficult to determine the parent distribution of the data set in advance. Marmolejo-Ramos and González-Burgos (2013) proposed a method that can assist in ameliorating this issue. These researchers recommend fitting the data with a set of potential parent distributions, and estimating which parent distribution gives the best fit. Once a parent distribution is identified, it could be possible to select an appropriate fitting normality test that is powerful against the type of distribution.

**FIGURE 2:**
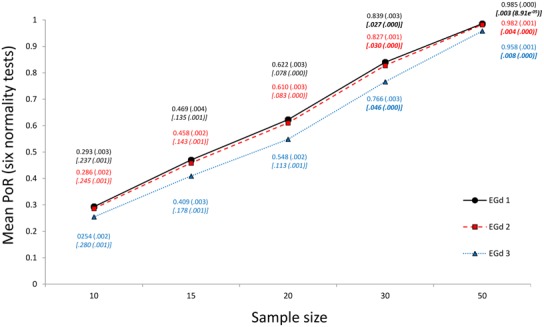
**Mean PoR (and ±1 SD) of a combined set of six normality tests for three EGds when *n* = 10, 15, 20, 30, and 50.** The associated mean *p*-values for each case, and their ±1 SDs (in parenthesis), are shown in italics and between brackets. *p*-values below 0.05 are bolded.

An alternative method in which the combined results of various normality tests are used is proposed herein. There are approximately 40 different types of normality tests (see Razali and Wah, 2010) that can be categorized as regression/correlations, empirical distribution functions, measure of moments, or a combination of these (see Romão et al., 2010; Marmolejo-Ramos and González-Burgos, 2013). New normality tests are still being proposed (e.g., Akbilgiç and Howe, 2011; Harri and Coble, 2011; He and Xu, 2013), which may lead to new categorizations. Thus, it is seems rather inadvisable to rely solely on one test, especially when considering that tests also differ based on the different characteristics of the normal distribution on which they focus (Romão et al., 2010). Therefore, a comprehensive assessment of normality would require the combination of results given by normality tests from different categories. That is, an average of the *p*-values given by normality tests belonging to the categories mentioned above should give an educated approximation of the normality of a given distribution. **Figure 2** shows the results of applying a normality-tests-combination method to the three EGds mentioned above when sample sizes are 10, 15, 20, 30, and 50 via the Marmolejo-Ramos and González-Burgos’ simulation method described above. The normality tests used were the SW, Shapiro–Francia (SF; these are regression/correlation-based tests), KS, Anderson–Darling (AD; these are empirical distribution function-based tests), Doornik–Hansen (DH), and the robust Jarque-Bera (rJB; these are measure of moments-based tests; details in relation to these tests can be found in Romão et al., 2010)^1^ .

This section aims to determine the degree of normality achieved by the outlier elimination and transformation methods described above, on various EGds. The six normality tests described above were used to provide a gage of the average level of normality achieved by the outlier methods. The simulation approach described above, which uses iterations of simulations and estimation of an average, was used in the study. In addition, the *p*-value approach described above was used to determine the PoRs after the outlier methods were applied to the EGds.

Three different sets of EGds were generated. The parameters were those described above: μ = 300, σ = 20, and τ = 300 (EGd1), μ = 400, σ = 20, and τ = 200 (EGd2), and μ = 500, σ = 20, and τ = 100 (EGd3). Each EGd was generated in four sample sizes: 15, 20, 30, and 50^2^. These parameters represent actual RT data and are taken from the 12 different EGds reported originally by Miller (1989). The simulation was carried out using the method proposed by Marmolejo-Ramos and González-Burgos (2013) and with the following parameters: *i* = 30, *s* = 20’000, and *a* = the Mean (and its ±1 *SD*). The mean *p-*value of the six normality tests described earlier was computed for each *s* product of the combination of EGd type, sample size, and outlier method. Across simulations, the PoR was the proportion of times the average *p*-values fell below, and were not equal to, an alpha level of 0.05. Finally, the mean PoR and *p-*value were estimated for each vector containing *i*.

Thus, for each combination of EGd, sample size, and outlier method, *i* number of PoR and *p-*value results were available. The mean results of the PoRs and *p*-values were submitted to a 3 (types of EGd = EGd1, EGd2, and EGd3) × 4 (sample sizes = 15, 20, 30, and 50) × 2 (outlier methods = transformation and elimination) ANOVA-type statistic (ATS; see Noguchi et al., 2012 for details) in order to determine main effects and interactions. The “type of EGds” was entered in the analysis as the between-subjects factor, while the other factors were entered as the within-subjects factors. The items for the outlier transformation method were the four transformation parameters of the Box–Cox transformations described above, i.e., -1, -0.5, 0, and 0.5 and the items for the outlier elimination method were the four methods discussed above, i.e., the MCD, ± 2 SD, ± 2.5 SD, and ± 3 SD methods. Comparisons of two dependent groups were performed via the Yuen test (*T_y_*; see Wilcox, 2012 for details).

In the particular case of the outlier elimination methods, the proportion of data eliminated (PoE) was estimated in the same way as the PoRs. That is, for each distribution to which an outlier elimination method was applied, the proportion of observations removed by a specific method was computed. Then, an average of PoE was estimated for the total number of simulations and each simulation run was iterated *i* times. Finally, the mean PoEs across iterations were computed.

**FIGURE 3:**
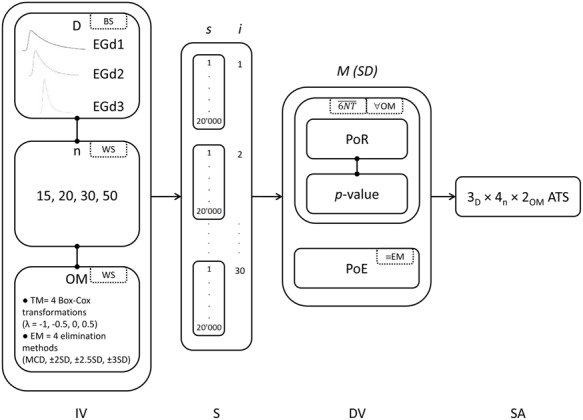
**Key components of the simulation study.** Specific details can be found in the ‘Materials and Methods’ section. IV, independent variables (BS, between-subjects factors; WS, within subjects factors; D, distributions; *n*, sample sizes, OM, outlier methods), S, simulation (*s,* number of simulations; *i*, number of iterations), DV, dependent variables [M (SD) = means and standard deviations; 6NT, mean across six normality tests; ∀OM, for all outlier methods; ≡EM, only for elimination methods], and SA, statistical analysis (ATS, ANOVA-type statistic).

Also, for both outlier methods, the mean *p-*value is reported in order to render more obvious the direct relationship between PoR and *p-*values. That is, the higher the mean *p-*value, the lower the PoR, and the lower the mean *p-*value, the higher the PoR. That is, more chances of normality rejection are paired with mean *p-*values, signaling non-normality. **Figure 3** illustrates the key features of the simulation study.

### A NOTE ON THE CHARACTERISTICS OF THE PRESENT SIMULATIONS

All throughout this article the simulation method used in the present study has been depicted so it is worthwhile emphasizing the value of the simulation approach used herein. Canonical simulation studies on normality test report tables of a unique number that represents the power of the test under study, i.e., the proportion of times the test rejected normality (here PoR) at the alpha level chosen. For instance, in an ideal simulation study in which 20’000 simulations are run, it is expected that in 1’000 of them the assumption of normality is incorrectly rejected when tested against a *N*(0,1), which in turn, gives a power of 0.05 (or a PoR of 0.05). However, if such simulation is run a second, third, or an *x* number of times, it is likely that the number of *N*(0,1) distributions flagged as non-normal, varies from 1000 every time. Therefore, giving a PoR of approximately 0.05. Such variation in the outcome can be due to several factors such as the type of RNG used, the use of seeding in the simulations, the statistical package used, and/or simply chance.

There is in fact another issue associated with the study of normality tests that can play a part in the simulation process. When a normality test is used against a *N*(0,1), a distribution of *x* number of observations, i.e., the number of simulations, containing the results of the test statistic is formed. CVs are then obtained by calculating the key quantiles of the test statistic’s distribution (e.g., the 95% quantile in positively skewed distributions when alpha is 0.05). However, the CVs found are directly dependent on the computation used to estimate the quantiles and there are various computations involved [for instance, the R software implements nine different quantile computations (see Hyndman and Fan, 1996)].

## RESULTS

### PROPORTION OF REJECTION AND *P*-VALUES

**FIGURE 4:**
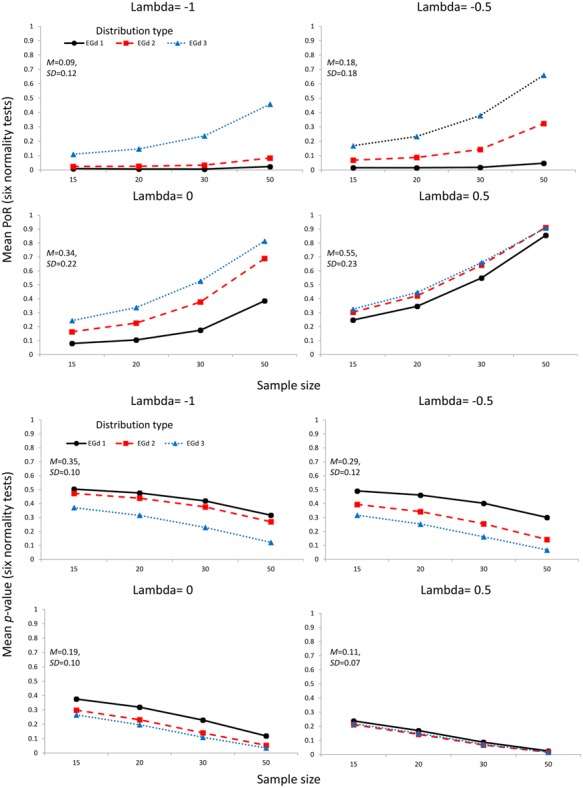
**Mean proportion of rejection (four **top** panels) and *p*-values (four **bottom** panels) of the outlier accommodation procedure via data transformation.** Lambda represents the parameter used to perform the transformation. Insets show the mean and SD estimates across distributions types and sample sizes.

**FIGURE 5:**
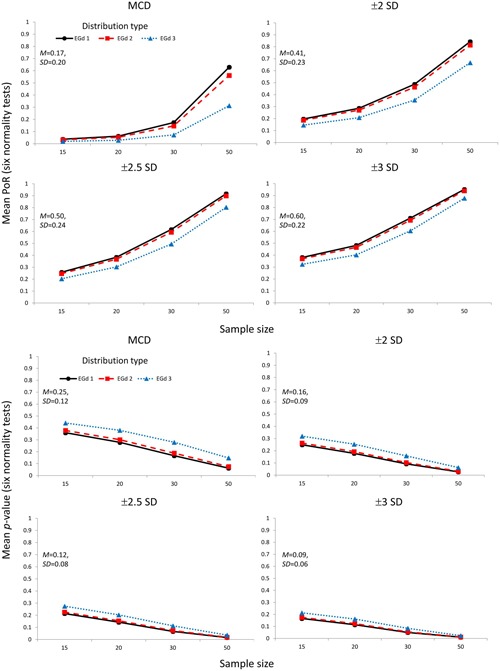
**Mean proportion of rejection (four **top** panels) and *p*-values (four **bottom** panels) of the outlier elimination procedures via the MCD and SD methods.** Insets show the mean and SD estimates across distributions types and sample sizes.

The mean PoR and mean *p*-values corresponding to the transformation of outliers via the Box–Cox transformation parameters are shown in **Figure 4**. **Figure 5** shows the mean PoR and mean *p*-values for the case of elimination of outliers via the MCD and the ±*n* SD methods.

**FIGURE 6:**
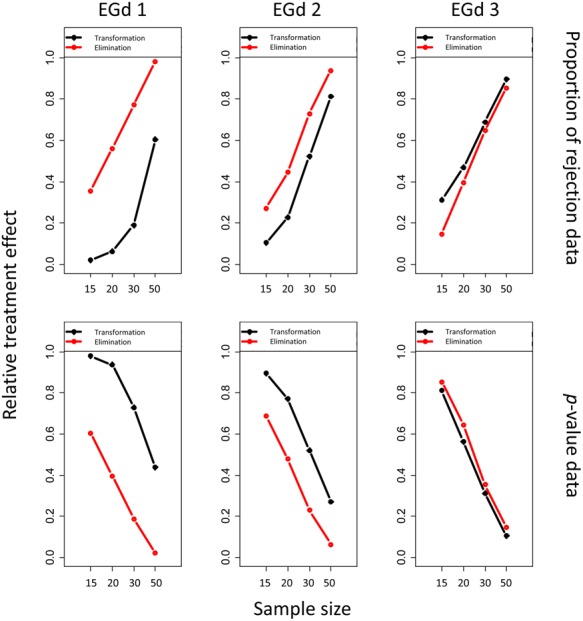
**Relative treatment effect plot of the three way interaction between distribution type, sample size, and outlier method for the proportion of rejection and *p*-value analyses**.

The ATS analyses suggested significant main effects of distribution type; sample size and outlier method and their two and three way interactions in both the PoR and *p*-value analyses (see **Table 1**). The effect sizes of the three way interactions are shown in **Figure 6**.

**Table 1:** Results of the ANOVA-type statistic (ATS).

DV	Main effect	Interaction
PoR	*F*_D_(1.94,82.53) = 4165.33, *p* < 0.001	*F*_D×O_(1.96,∞) = 22335.40, *p* < 0.001
	*F*_O_(1,∞) = 31857.81, *p* < 0.001	*F*_S×O_(2.72,∞) = 883.00, *p* < 0.001
	*F*_s_(2.54,∞) = 78415.56, *p* < 0.001	*F*_D×S_ (4.90,∞) = 525.59, *p* < 0.001 *F*_D×O×S_ (5.17,∞) = 267.56, *p* < 0.001
*p*V	*F*_D_(1.93,81.51) = 1681.45, *p* < 0.001	*F*_D×O_(1.96,∞) = 34226.12, *p* < 0.001
	*F*_O_(1,∞) = 74198.60, *p* < 0.001	*F*_S×O_(2.85,∞) = 502.25, *p* < 0.001
	*F*_s_(2.82,∞) = 82610.79, *p* < 0.001	*F*_D×S_(5.50,∞) = 498.73, *p* < 0.001 *F*_D×O×S_(5.57,∞) = 255.15, *p* < 0.001
PoE	*F*_D_(1.96,83.66) = 4382.35, *p* < 0.001	*F*_D×E_(5.02,∞) = 165.09, *p* < 0.001
	*F*_E_(2.66,∞) = 267304.12, *p* < 0.001	*F*_S×E_(6.15,∞) = 4529.77, *p* < 0.001
	*F*_s_(2.82,∞) = 186.08, *p* < 0.001	*F*_D×S_(5.42,∞) = 12.11, *p* < 0.001 *F*_D×E×S_(11.18,∞) = 35.62, *p* < 0.001

*DV, dependent variable; PoR, proportion of rejection data; *p*V, *p-*value data; PoE, proportion of elimination data; D, distribution type (EGd1, EGd2, and EGd3); O, outlier method (elimination and transformation); S, sample size (15, 20, 30, and 50), and E, elimination method (MCD, ±2 SD, ±2.5 SD, and ±3 SD).*

As shown in the first row in **Figure 6**, the likelihood of the rejection of normality increases as sample size (i.e., from 15 to 50) and distribution skewness increase (i.e., from EGd3 to EGd1). This result is corroborated by the *p*-values analyses in that as sample size and distribution skewness increase, the *p*-values decrease (second row in **Figure 6**). This is a phenomenon recognized in research on the power of normality tests (see Marmolejo-Ramos and González-Burgos, 2013) and is replicated here by the main effects of sample size and distribution type (see **Table 1**). An interesting result from the relative treatment effects plots, however, is that while the likelihood of rejection of normality increases from EGd3 to EGd1 in the case of elimination methods, an opposite pattern occurs to the transformation methods. This result indicates that transformation methods have greater normalization power than elimination methods as the distribution becomes more skewed. The relative treatment effects plots for the *p*-value data corroborate this by showing that transformation methods lead to higher *p*-values than elimination methods as the distribution becomes more skewed.

As **Figures 4 and 6** indicate, the transformation methods seem to lead to decreased normality rejection as compared with elimination methods. The larger effects of the former methods over the latter are summarized in **Figure 6**. As shown in **Figure 4**, the transformation methods seem to be particularly useful when dealing with highly skewed distributions (i.e., EGd1) in that, across sample sizes, low PoRs, and high *p*-values were obtained for these distributions after transformation. Specifically, the results indicate that transformations with lambda -1 would seem to provide the best results (see insets in **Figure 4**). These results are in agreement with past research suggesting that the inverse transformation has a strong normalization effect (see Ratcliff, 1993).

### PROPORTION OF ELIMINATION

**FIGURE 7:**
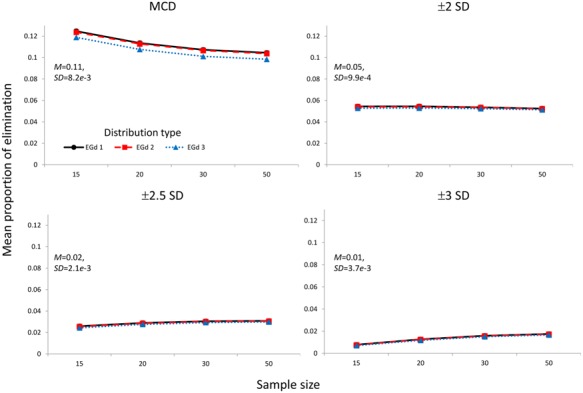
**Mean proportion of elimination of the outlier elimination procedures via the MCD and SD methods.** Insets show the mean and SD estimates across distributions types and sample sizes.

The mean PoE corresponding to the elimination of outliers via the MCD and ±*n* SD methods is shown in **Figure 7**.

**FIGURE 8:**
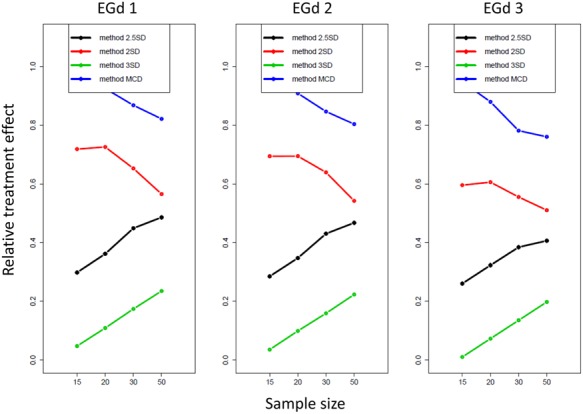
**Relative treatment effect plot of the three way interaction between distribution type, sample size, and outlier method for the proportion of elimination analyses**.

The ATS analyses suggested significant effects of distribution type; sample size, and outlier elimination method and their two and three way interactions in the PoE analyses (see **Table 1**). The effect sizes of the three way interactions are shown in **Figure 8**.

**Figure 5** reports the mean proportion of rejection achieved by each method for each distribution in different sample sizes and their associated *p*-values. The results suggest that the MCD method seems to lead to lower PoR and higher *p*-values than the SD methods. However, by comparing **Figures 5 and 7**, a trade-off between the PoRs (and associated *p*-values) and the POE associated with each of these methods becomes clear. Thus, while the MCD method leads to the lowest PoRs, it does have the highest POE. On the contrary, the ±3 SD method has low PoE but at the cost of leading to a rather high proportion of normality rejection.

As the relative treatment effect plot in **Figure 8** indicates, for all methods, the likelihood of eliminating more data increases as the distribution becomes more skewed; i.e., from EGd 3 to EGd 1. However, while the MCD and ±2 SD’s likelihood of rejecting data appears to reduce as sample size increases, for the remaining methods such a likelihood increases as sample size increases.

In summary, the key result is that the transformation methods are more effective than the elimination methods at normalizing positively skewed distributions. That is, the outlier method had a main effect. Thus, indicating that across distributions types and sample sizes the transformation methods led to lower PoR (*M*_PoR_ = 0.29, SD = 0.17) and higher *p*-values (*M_p_*_-value_ = 0.24, SD = 0.09) than the elimination methods (*M*_PoR_ = 0.42, SD = 0.22; *M_p_*_-value_ = 0.16, SD = 0.09) [PoR: *T_y_* (359) = -14.54, *p* < 0.001; *p*-value: *T_y_* (359) = 20.26, *p* < 0.001].

## DISCUSSION

The results of this simulation study suggest that the Box–Cox transformation methods outperform the elimination methods in normalizing positively skewed data and the more skewed the distribution, the more effective the transformation methods in normalizing such type of data. Various ideas need to be discussed in relation to this finding.

The difference between transformations and elimination procedures is that transformations seek to stabilize variance and skewness (see Bartlett, 1947) whereas the other procedures are devised to eliminate extreme observations; as a by-product, both methods help in improving normality. However, these methods ultimately aim to determine the best estimator of central tendency. One could argue that these procedures simply distort the original data sets in order to render them suitable for a parametric test. Although there are arguments in favor of using parametric tests regardless (see Schmider et al., 2010), others advocate the use of other statistical methods (e.g., Wilcox, 2012). For instance, Lachaud and Renaud (2011) indicate that either the data should be filtered (e.g., via the ±*n* SD approach) before analysis using general linear modeling (e.g., ANOVA, quasi-*F*, and multilevel modeling) or analyzed using robust methods (e.g., ATS, bootstrap, and permutation methods). These authors also give useful recommendations as to how to analyze data when using general linear modeling approaches. Hence, a combination of the methods studied here with robust techniques could also be productive (Rashid et al., 2013, term this approach ‘side-by-side analyses’). Some researchers have taken these methods further. For instance, Ulrich and Maienborn (2010) took the mean RT of correct trials for each subject in each condition and compared the results with those obtained when the median RT and the 10% trimmed mean of correct trials for each subject in each condition were taken. That is, the researchers compared the results of analysis using a 0 (arithmetic mean), 10, and 50% (median) trimmed means. Finally, they performed analyses on the means of the trimmed means. Other researchers opt for taking the median RT of correct trials for each subject in each condition and perform analyses on the means of those medians (see for example Ansorge et al., 2010; Rein and Markman, 2010). Trimmed means, and other robust estimators of central tendency (e.g., Rosenberg and Gasko, 1983; Wilcox and Keselman, 2003; Bickel and Frühwirth, 2006; Vélez and Correa, 2014), can therefore be seen as non-invasive forms of data elimination in that outlying observations are temporarily canceled out in order to estimate an average.

Applying the methods studied herein to data believed to be non-normal, does not automatically guarantee that the data has met parametric assumptions. That is, it is important to corroborate, via graphical and formal tests, that these assumptions have been reached. Although this is a well-known recommendation, it is rare to find published papers reporting normality or homogeneity tests in order to justify the use of parametric analyses. It is therefore important that whatever method is used to filter data, formal normality, and homogeneity tests are reported in order to substantiate the use of parametric tests.

### METHODOLOGICAL CONSIDERATIONS AND FUTURE STUDIES

Every research study has room for improvement and this study is no exception. Admittedly, the estimation of PoR and *p*-values used here is rather liberal and may have some degree of Type I error attached to it. Thus, a replication study could estimate CVs for each normality test employed via quantiles as is traditionally done (although see section 2.2) and *p-*values could be combined via conservative methods such as the Stouffer method (see Vélez et al., under review). Also, other normality tests that are particularly robust to the distributions being studied could be considered. Equally important, other distributions that are a good fit for real data should be included in the simulation study. For instance, in the case of RT, data distributions such as Weibull and Log-Normal need to be studied. Another type of data commonly encountered in experimental research but less studied, is that of discrete *n*-point Likert-type data. Distributions that fit this type of data could be studied in the context of outliers and normality research as well. Yet, the studies suggested here should be preceded by research demonstrating how well various potential candidate distributions fit RT and Likert-type data (e.g., via AIC measures) and showing which distributions seem to give the best fits in both real and simulated RT and Likert-type data. Indeed, there should be research aimed at grounding the parameters of distributions fitting RT and Likert-type data into psychological processes of interest (e.g., McAuley et al., 2006 explained the parameters of the EGd in terms of cognitive processed tapped via RTs). To the best of our knowledge such research is yet to be done.

Although some of the most commonly employed outlier elimination and transformation methods were addressed herein, other methods should also be studied. For instance, data truncation and outlier replacement are procedures also found in papers reporting experimental results in cognitive science (an example of data truncation can be found in Bub and Masson, 2010; an example of outlier replacement can be seen in Pylyshyn and Storm, 1988). The performance of newer methods such as the Ueda (1996/2009), van der Loo (2010), and ±2.5 MAD (Leys et al., 2013) should be studied in the context of RT data.

Finally, it can be contended that in principle, the procedures studied here should not only improve data’s normality but also their homogeneity. Thus, future studies should test the effects of the procedures studied here on the homogeneity of two or more batches of data. Canonical tests such as the Levene and the Brown-Forsythe test and recent robust versions of them (e.g., De Almeida et al., 2008) should be used to verify this claim.

## CONCLUSION

This paper sets out to offer an educated consensus on the recommended approach in cases where data need to be treated in order to submit to a parametric test. The results indicate that methods that transform the data in order to accommodate outliers lead to higher chances of normalization than methods that eliminate data points. Although some of the most commonly used elimination and transformation methods were studied herein, further methods need to be considered. Other distributions that can be used to model reaction time and Likert-type data should also be addressed in future studies.

## AUTHOR CONTRIBUTIONS

Fernando Marmolejo-Ramos thanks Delphine Courvoisier and Firat Özdemir for their help with earlier versions of this project. Fernando Marmolejo-Ramos also thanks Kimihiro Noguchi for his help with his ‘nparLD’ R package and Xavier Romão for facilitating access to HPC facilities. Finally, the authors thank Robyn Groves and Rosie Gronthos for prooreading this manuscript.

## Conflict of Interest Statement

The authors declare that the research was conducted in the absence of any commercial or financial relationships that could be construed as a potential conflict of interest.

## Footnotes

1 All of these tests are implemented in R in the following packages: “stats” package (SW test), “nortest” package (AD, KS, and SF tests), “lawstat” package (rJB test), and “normwhn.test” package (DH test). Note that the *p*-values computed for the SW test are based on the Royston method (Royston, 1982a,b, 1995), the version of the KS test in the “nortest” package is the Lilliefors, the critical values used to compute the rJB are obtained by an approximation to a χ^2^ distribution and with zero number of Monte Carlo simulations for the empirical critical values, and the *p*-value registered for the DH test was that labeled “sig.Ep” in the output.

2 A pilot simulation showed that EGds of sample size 10 were in some cases trimmed down to seven when the elimination methods were used. Because such a low sample size affected the results of the AD and the DH tests, the sample size 10 was excluded from the final simulation study.
